# Role of distinct surfaces of the G9a ankyrin repeat domain in histone and DNA methylation during embryonic stem cell self-renewal and differentiation

**DOI:** 10.1186/1756-8935-7-27

**Published:** 2014-10-22

**Authors:** Danielle Bittencourt, Brian H Lee, Lu Gao, Daniel S Gerke, Michael R Stallcup

**Affiliations:** 1 From the Department of Biochemistry and Molecular Biology, Norris Comprehensive Cancer Center, University of Southern California, NOR 6314, 1441 Eastlake Avenue, Los Angeles 90089-9176, CA, USA

**Keywords:** Histone methylation, DNA methylation, Embryonic stem cell, G9a, Ankyrin, H3K9, DNMT3A, Oct3/4

## Abstract

**Background:**

Epigenetic modifications such as histone and DNA methylation are essential for silencing pluripotency genes during embryonic stem cell (ESC) differentiation. G9a is the major histone H3 Lys9 (H3K9) methyltransferase in euchromatin and is required for the *de novo* DNA methylation of the key regulator of pluripotency *Oct3/4* during ESC differentiation. Surprisingly, the catalytic activity of G9a is not required for its role in *de novo* DNA methylation and the precise molecular mechanisms of G9a in this process are poorly understood. It has been suggested that the G9a ankyrin repeat domain, which can interact with both H3K9me2 and the DNA methyltransferase DNMT3A, could facilitate *de novo* DNA methylation by bridging the interaction between DNMT3A and H3K9me2-marked chromatin.

**Results:**

Here, we demonstrate that the G9a ankyrin domain H3K9me2-binding function is not required for the *de novo* DNA methylation of *Oct3/4* during ESC differentiation. Moreover, we show that the interaction between the G9a ankyrin domain and DNMT3A is not sufficient to ensure efficient *de novo* DNA methylation. More importantly, we characterize a specific residue of the G9a ankyrin domain (Asp905) that is critical for both maintaining cellular H3K9me2 levels in undifferentiated ESCs and for the establishment of *de novo* DNA methylation during differentiation.

**Conclusions:**

These results represent an exciting breakthrough, which reveals 1) an unexpected critical biological function of the G9a ankyrin domain in global histone H3K9 methylation and 2) valuable insights into the molecular mechanisms and interaction surfaces through which G9a regulates *de novo* DNA methylation of *Oct3/4* during ESC differentiation.

## Background

The remarkable property of embryonic stem cells (ESC) to remain in a pluripotent state, poised to execute a broad range of developmental programs, is due to fine control of proper gene expression patterns by key regulators of pluripotency such as the Oct3/4 and Nanog transcription factors. This pluripotent state is characterized by maintenance of transcriptionally permissive chromatin, which underlies high genome plasticity from which other gene expression patterns are derived during development [1-4]. Epigenetic modifications such as histone and DNA methylation are essential for chromatin reorganization during development, resulting in the silencing of early embryonic genes and activation of lineage-specific genes that drive differentiation in cell fates [5].

**Figure 1:**
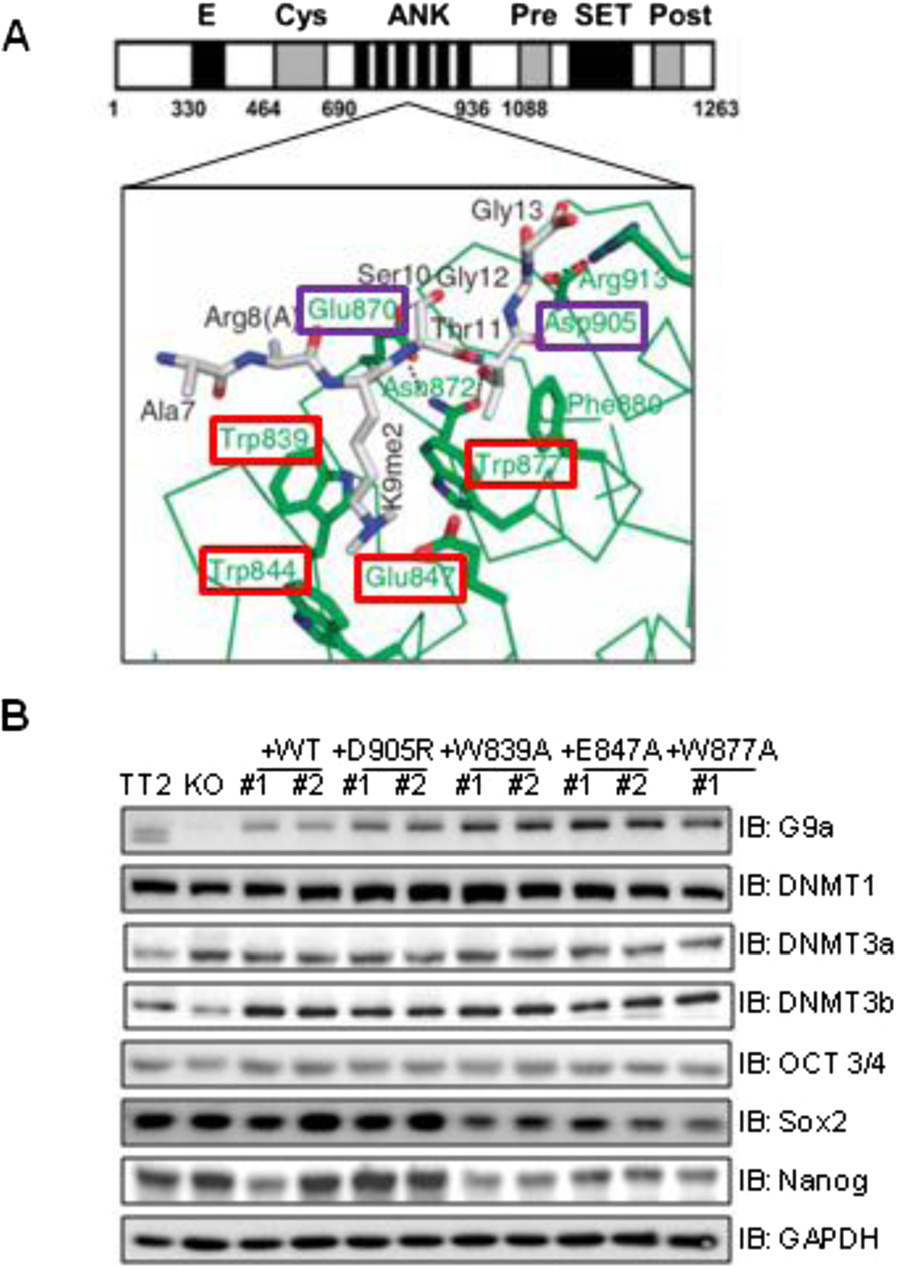
**Characterization of mouse embryonic stem (ES) clonal cell lines expressing G9a bearing point mutations in the cage and non-cage residues of the ankyrin repeat (ANK) domain. (A)***Top panel:* Diagram of full-length mG9a showing amino acid sequence numbers and specific domains: *E*, Glu-rich; *Cys*, Cys-rich ring finger-like; *ANK*, ankyrin repeat; *SET*, methyltransferase; and *Pre* and *Post*, Cys-rich Pre-SET and post-SET. *Bottom panel:* Schematic representation of the structural basis for ankyrin repeat (green) recognition of dimethylated histone H3 Lysine 9 (gray). G9a residue numbers are shown. H3K9me2 binds in a partial hydrophobic cage composed of residues highlighted in red. Peptide binding is further specified by the interaction of non-cage G9a residues (highlighted in purple) with H3 Ser10, Thr11, Gly12 and Gly13. **(B)** Immunoblots (*IB*) were performed on the indicated proteins (with GAPDH as loading control) using whole cell extracts derived from wild-type (TT2) mouse embryonic stem cells (mESCs), *G9a*-null mESCs (knock-out; KO) or distinct clonal ESC lines (indicated by #1 and #2) stably expressing wild-type G9a (+WT) or G9a harboring the indicated point mutations in the ANK domain.

Underlining the importance of histone and DNA methylation in the control of proper gene expression patterns in ESCs, disruption of the *G9a* histone methyltransferase gene in mice results in growth retardation and early embryonic lethality due to deregulation of developmental genes [6]. *G9a*-null ESCs display a dramatic reduction in cellular mono- and dimethylated histone H3 lysine K9 (H3K9) levels, indicating its role as the major histone H3K9 methyltransferase. Furthermore, *G9a*-null ESCs show deficient *de novo* DNA methylation of several early embryonic genes, including *Oct3/4* and *Nanog* during differentiation [7-9], suggesting a key role of G9a in establishing *de novo* DNA methylation. However, in contrast to histone methylation, the catalytic function of the G9a C-terminal SET domain (Figure 1A, top panel) is not required for its role in *de novo* DNA methylation [7,10].

A recent report has shown that the deletion of the G9a central ankyrin repeat domain (ANK, Figure 1A, top panel) results in impaired *de novo* DNA methylation of the *Oct3/4* gene promoter during ESC differentiation [7]. Interestingly, the G9a ANK domain also binds to the *de novo* DNA methyltransferase DNMT3A *in vitro*[7], although it has not yet been determined whether this interaction is required and/or sufficient for G9a-mediated *de novo* DNA methylation. Furthermore, the G9a ANK domain specifically binds to the product of G9a catalytic activity: histone H3 mono- or dimethylated at K9 [11]. Indeed, G9a binding to K9-methylated histone H3 involves recognition of two different features of the methylated histone by a discreet region of the ANK domain, as previously demonstrated by X-ray crystallography: 1) a partial hydrophobic cage of ANK recognizes the dimethylamino moiety of H3K9me2, and 2) a nearby non-cage functional surface of ANK interacts with residues 10 to 13 of histone H3 [11] (Figure 1A, bottom panel). However, the biological function of this binding is not fully understood, and to date, the precise molecular mechanisms by which G9a and, in particular its ANK domain, contribute to *de novo* DNA methylation remain unclear.

To address these questions, we established clonal ESC lines expressing G9a with point mutations in the ANK domain that prevent K9-dimethylated histone H3 binding. With these cell lines, we investigated the role of this histone H3 binding surface in regulating markers of ESC differentiation, global cellular H3K9 methylation, and *de novo* DNA methylation of the *Oct3/4* gene promoter both in undifferentiated ESCs and during retinoic acid (RA)-induced differentiation. Our results reveal that these three parameters of ESC biology have distinct requirements for different surfaces of G9a. Surprisingly, we found that different point mutations that disrupt ANK domain binding to K9-dimethylated histone H3 had different phenotypes, suggesting that the histone H3 binding surface of the ANK domain may have multiple functions involving multiple interacting protein partners. We also define a specific residue in the G9a ANK domain that is critical for its role in *de novo* DNA methylation. Altogether, our findings represent an exciting breakthrough, which can reveal 1) novel biological functions of the G9a ANK domain, and 2) valuable insights into the mechanisms of the distinct functional surfaces of the G9a ANK in supporting *de novo* DNA methylation, H3K9 methylation and ESC differentiation.

## Results

### Derivation of clonal embryonic stem cell lines expressing G9a with point mutations in the ankyrin repeat domain

Our previous X-ray crystallography studies characterized six residues of the G9a ankyrin repeat (ANK) domain that are involved in G9a binding to the N-terminal tail of histone H3 that is mono- or dimethylated at K9 [11]. While four of these residues (Trp839, Trp844, Glu847 and Trp877) form a partial hydrophobic cage that interacts with the dimethylamino moiety of H3K9me2 (Figure 1A, bottom panel, highlighted in red), residues Glu870 and D905 of the G9a ANK non-cage functional surface interact with amino acids 10 to 13 of histone H3 [11] (Figure 1A, bottom panel, highlighted in purple). A single point mutation in any of these six G9a residues is sufficient to disrupt the interaction *in vitro* between G9a and a histone H3 N-terminal peptide dimethylated at K9. Importantly, none of these mutations abolish G9a catalytic function as determined by *in vitro* assays or its structural integrity, as demonstrated by the ability of the G9a ANK domain containing any of these mutations to interact with protein partners GRIP1 and Gfi1 [11]. Thus, to date the biological function of the histone H3 interacting surface of the G9a ANK domain is unknown.

**Figure 2:**
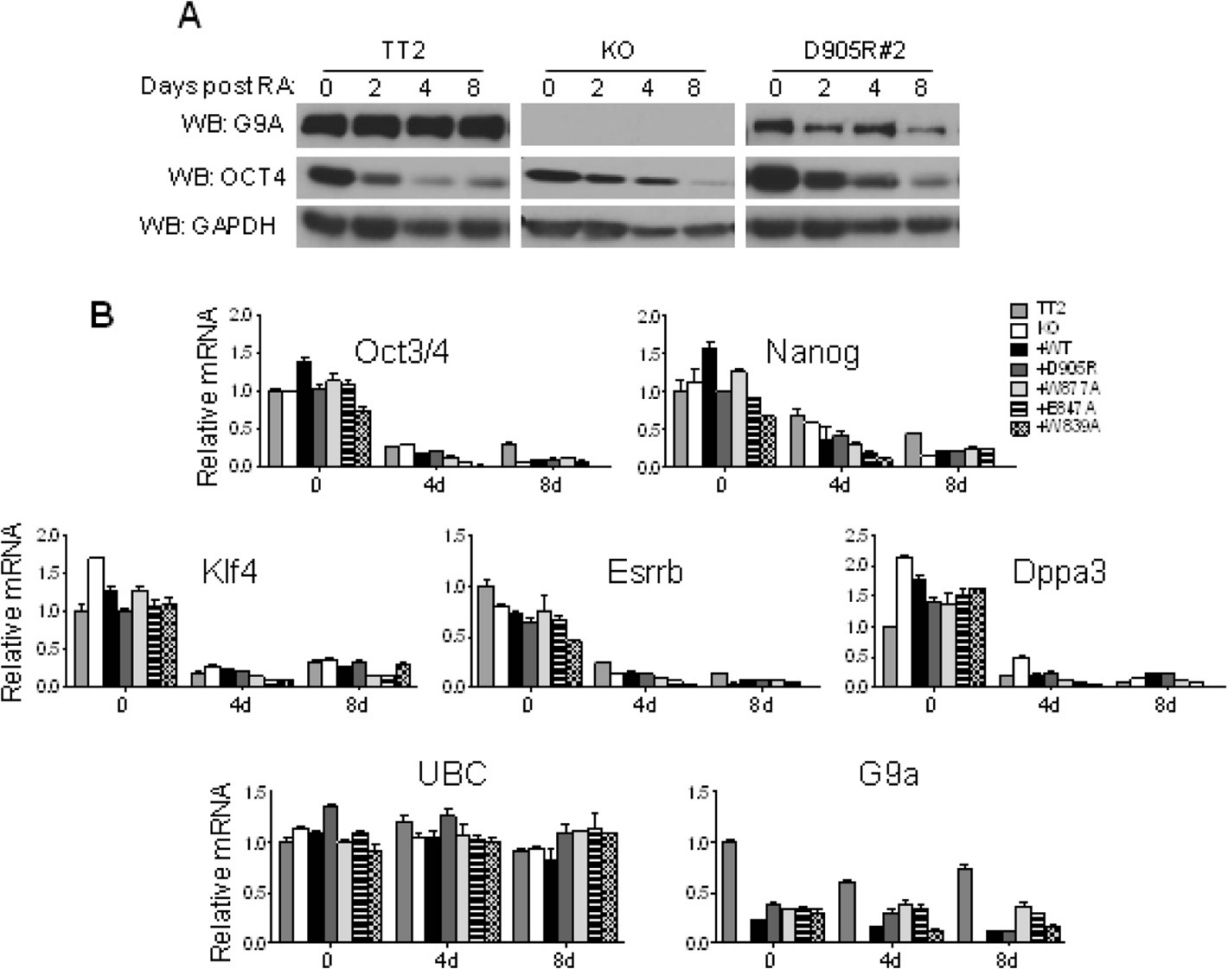
**G9a is not required for the transcriptional repression of selected early embryonic genes during retinoic acid (RA)-induced differentiation.** The indicated mESC lines were untreated (0) or treated with 1 μM RA for 2, 4 or 8 days. **(A)** Immunoblots were used to determine G9a, Oct3/4 and GAPDH (loading control) protein levels in whole cell extracts. **(B)** Graph shows the messenger (mRNA) levels for the indicated genes using RT-qPCR analysis with *Ubiquitin C (UBC)* as the normalization control. All mRNA levels are normalized to the TT2 0 RA sample. Results shown are mean ± SD for three PCR reactions performed on the same cDNA sample and are representative of three independent experiments.

In order to investigate whether the methylhistone H3 binding function of the G9a ANK domain is required for mESC differentiation and the accompanying *de novo* DNA methylation of key pluripotency genes, *G9a*-null mESCs (knock-out; KO) were used to generate stable clonal cell lines expressing either wild-type (+WT) G9a or G9a containing various ANK domain point mutations mentioned above. Importantly, two independent clones expressing each G9a mutant were established in order to control for potential clonal artifacts. Immunoblots were performed to verify that the G9a expression level in these cell lines is comparable to the endogenous G9a expression in the wild-type parental mESC line TT2 (Figure 1B, top panel). Importantly, the protein expression levels of master regulators of pluripotency such as Oct3/4, Sox2 and Nanog (Figure 1B), as well as of key players in DNA methylation (DNA methyltransferases: DNMT3A, DNMT3B and DNMT1), were also comparable in all undifferentiated ESC lines (Figure 1B). Immunoblots were repeated several times using different protein extracts and slight differences observed in protein expression levels between cell lines seen here (Figure 1B) were not found consistently. Notably, messenger RNA (mRNA) expression levels of early embryonic genes (*Oct3/4, Nanog, Klf4, Esrrb* and *Dppa3*) determined by quantitative RT-qPCR were similar in all cell lines (Figure 2B).

### A specific histone H3 binding surface of the G9a ankyrin repeat domain is required for establishing *de novo* DNA methylation during mouse embryonic stem cell differentiation

**Figure 3:**
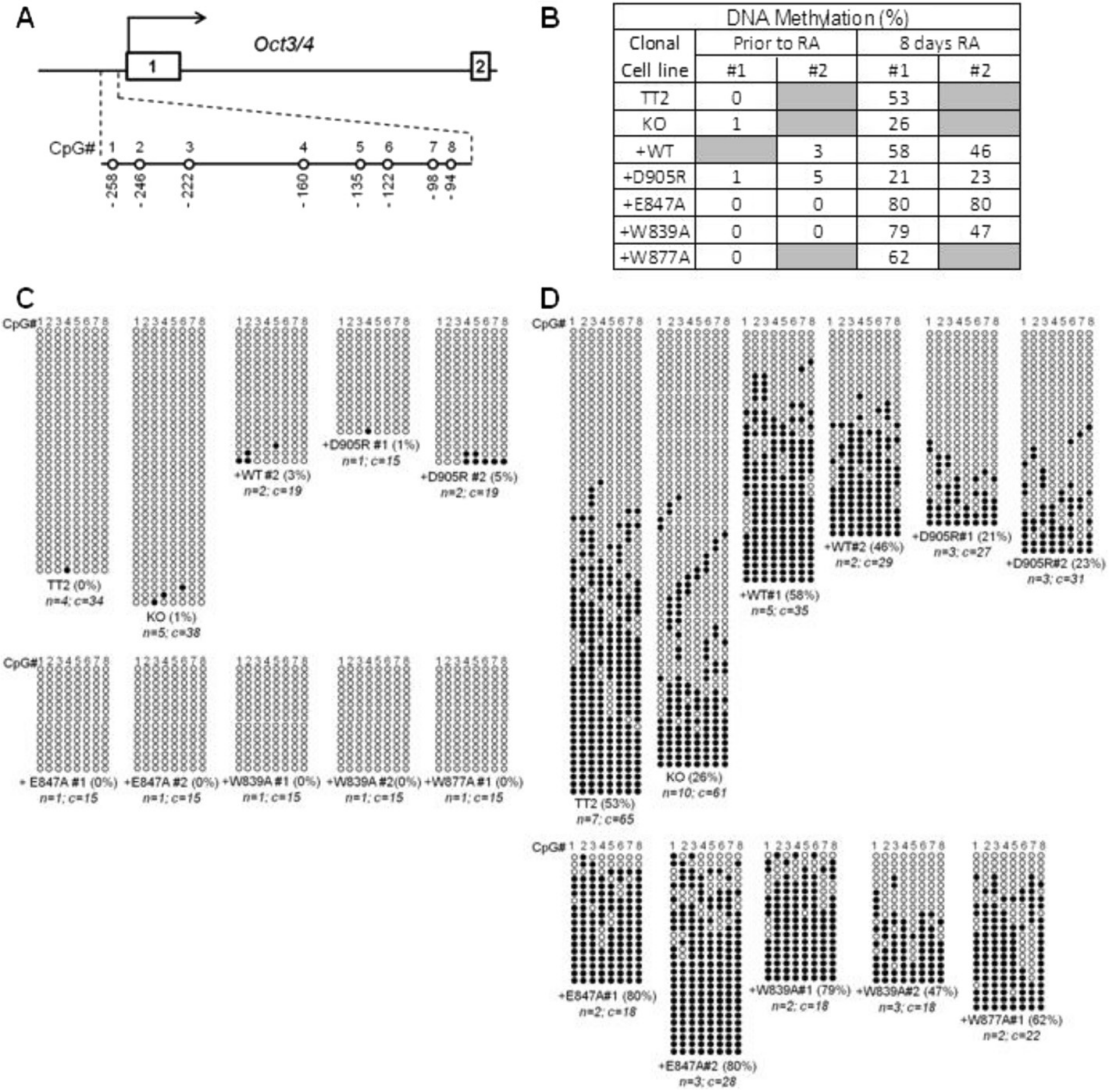
**The G9a non-cage histone H3 binding surface of the ankyrin repeat (ANK) domain is required for *de novo *DNA methylation of the *Oct3/4 *gene promoter during retinoic acid (RA)-induced embryonic stem cell (ESC) differentiation.** DNA extracted from the indicated mouse embryonic stem cell (mESC) lines, grown under self-renewal conditions or treated with RA for 8 days to induce differentiation, was treated with sodium bisulfite, amplified using specific *Oct3/4* promoter primers, cloned and subjected to sequence analysis. **(A)** Schematic diagram of CpG positions within the *Oct3/4* gene promoter is shown. The methylation status of eight CpG sites (no. 1 to 8) within the promoter (-90 to -258 relative to the transcription start site) was examined. **(B)** Percentage of DNA methylation for the *Oct3/4* promoter region encompassing eight CpG sites of each clonal cell line is indicated in the table, which summarizes data shown in **(C)** and **(D)**. DNA extracted from the indicated mESC lines that were untreated **(C)** or treated with RA for 8 days **(D)** was treated with sodium bisulfite, amplified using specific *Oct3/4* promoter primers, cloned and subjected to sequence analysis. Each horizontal row represents results from sequencing a separate DNA clone. The overall percentage of methylated CpG sites is indicated in parentheses beside the clone designation. The number of independent biological replicate experiments (*n*) from which the total number of DNA clones (*c*) were derived is also indicated.

Several reports demonstrate the requirement of G9a and its ANK domain for proper *de novo* DNA methylation of numerous early embryonic genes [6-8,12]. However, the molecular mechanisms of G9a ANK domain function in facilitating DNA methylation remain unknown. Using the ESC clones expressing the G9a point mutants described above, we investigated the role of the methyl-histone binding surface of the G9a ANK domain in the *de novo* DNA methylation of the *Oct3/4* gene promoter during ESC differentiation. Differentiation of stable ESC lines expressing wild-type or mutant G9a was induced by the addition of retinoic acid (RA) to the growth medium. The DNA methylation status of the *Oct3/4* proximal promoter region prior to and after 8 days of RA treatment (Figure 3) was assessed by sequencing PCR products generated from bisulfite-treated genomic DNA. As expected, in undifferentiated mESCs, the *Oct3/4* gene promoter is not methylated (Figures 3B and 3C). In agreement with previous findings, mESCs expressing either endogenous wild-type G9a (TT2) or exogenously introduced wild-type G9a (+WT) underwent *de novo* DNA methylation of the *Oct3/4* promoter, with CpG methylation levels of 46 to 58% in the region examined after 8 days of RA treatment (Figures 3B and 3D, lower panel). Point mutations in key hydrophobic cage residues of the G9a ankyrin domain that specifically recognize the dimethylamino moiety of H3K9me2 (+W839A, +W877A and + E847A) [11] did not compromise *de novo* DNA methylation of *Oct3/4* (Figure 3B and 3D). These results suggest that G9a binding to histone H3 containing mono- and dimethyl K9 is not required for *de novo* DNA methylation. In contrast, mESCs expressing G9a harboring a single point mutation of the Asp905 residue located in a distinct non-cage surface of the ANK domain that interacts with histone H3 residues 10 to 13 [11] display more than a twofold reduction in *de novo* DNA methylation levels of *Oct3/4*, similar to the levels observed in *G9a*-null mESCs (knock-out; KO) (Figure 3B and 3D). Although some degree of clonal variability was observed between the two W839A mutant clones, both clones have higher DNA methylation levels after 8 days of RA treatment compared to the KO and D905R. Therefore, we can conclude that the W839A mutation does not impair *de novo* DNA methylation of Oct4. In summary, these results indicate that the cage and non-cage portions of the dimethyl-histone H3 binding surface have common and distinct functions, that is, binding of histone H3 containing dimethyl K9 and regulation of *de novo* DNA methylation, respectively.

### The G9a ankyrin repeat domain, but not its histone-binding surfaces, is required for the interaction between G9a and DNMT3A

**Figure 4:**
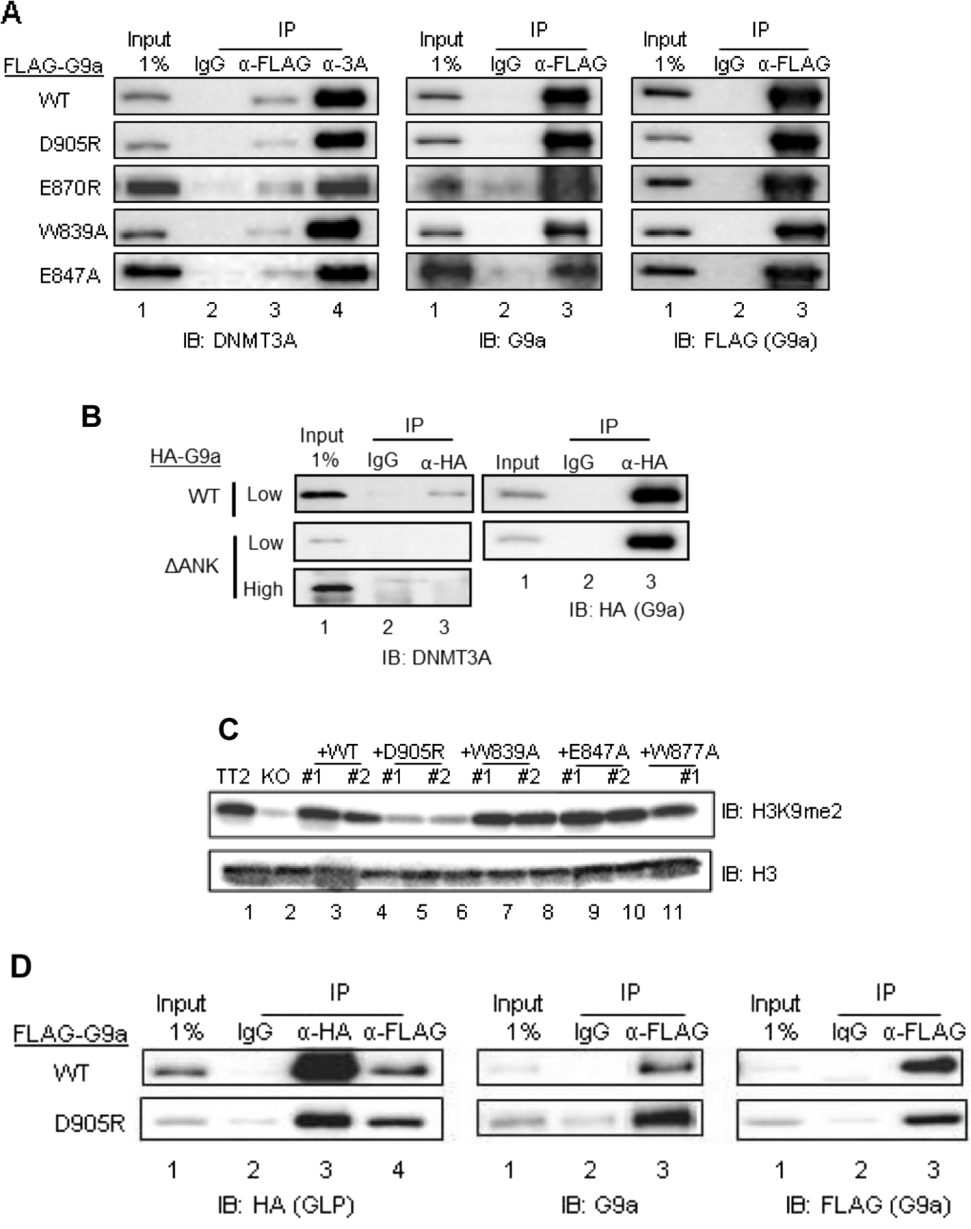
**Mutations in the histone H3 binding surface of the G9a ankyrin repeat (ANK) domain do not abrogate binding to DNMT3A.** COS- 7 cells were transiently transfected with pcDNA3.1-mDNMT3A **(A and B)** or pSG5-HA-mGLP full-length **(D)** and pSG5-FLAG-mG9a full-length **(A, B and D)** or pSG5-HA-mG9a.ΔANK **(B)**. G9a was either wild-type (WT) or contained the indicated point mutations in the ANK domain. G9a, GLP and DNMT3A were immunoprecipitated from cell extracts with an anti-FLAG/anti-HA, anti-HA or anti-DNMT3A antibody, respectively. Non-immune IgG antibody was used for immunoprecipitation background estimation. Bound proteins were analyzed by immunoblot (*IB*) with the indicated antibodies. A 1% input sample was loaded for comparison. High (3 minutes) and low (1 minute) exposure times are shown **(B)**. **(C)** Immunoblots were used to determine H3K9me2 and H3 protein levels in acid histone extracts from the indicated undifferentiated mESC lines.

Previous studies have shown that the G9a ANK domain is required *in vitro* for binding between G9a and DNMT3A [7]. We confirmed these findings via co-immunoprecipitation of transiently transfected human influenza hemagglutinin (HA) tagged full-length WT or ΔANK mutant mouse G9a and full-length mouse DNMT3A in a different cell line (COS-7 cell extracts) (Figure 4B). Although the interaction with DNMT3a is observed with WT G9a, it is abrogated in the G9a ΔANK mutant (Figure 4B, left panel, lane 3). However, the specific functional surface of the G9a ANK domain responsible for this binding, as well as the physiological relevance of this interaction for establishing *de novo* DNA methylation, has not been defined. In order to determine whether the impaired *de novo* DNA methylation observed in the D905R non-cage G9a ANK mutant ESC lines is due to compromised interaction between DNMT3A and the mutant G9a, co-immunoprecipitation of transiently transfected FLAG-tagged full-length (WT or mutant) mouse G9a and full-length mouse DNMT3A was performed in COS-7 cell extracts. As expected, co-immunoprecipitated DNMT3A was detected when antibodies against FLAG were used for immunoprecipitation of wild-type G9a (Figure 4A, left panel, lane 3). Interestingly, none of the mutations in the G9a ANK domain eliminated binding between G9a and DNMT3A (Figure 4A, left panel lane 3). Anti-FLAG and Anti-G9a immunoblots on the same samples (Figure 4A, right and middle panels, respectively) are shown as a control for G9a immunoprecipitation. Although the efficiency of DNMT3A co-precipitation varied among the clones, the pattern of variation did not correlate with the observed DNA methylation of the *Oct3/4* promoter; that is, DNMT3A co-precipitation with the D905R mutant (which has reduced DNA methylation of *Oct3/4*) is equal to or better than that for several of the other mutants (which support wild-type DNA methylation of *Oct3/4*). These results suggest that G9a binding to DNMT3A via the G9a ANK domain does not involve the ANK histone H3 binding surface but rather a different part of the ANK domain. More importantly, these data show that, although G9a and DNMT3A interaction is maintained in the D905R non-cage ANK mutant, this interaction is not sufficient to ensure proper *de novo* DNA methylation and a non-redundant function of the non-cage surface of the ANK H3 binding domain is critical to this process.

### The G9a non-cage ankyrin repeat domain histone-binding surface is required to maintain global cellular H3K9me2 levels

The G9a C-terminal SET catalytic domain is responsible for its H3K9 methyltransferase activity [13]. As shown previously [6], we found that global H3K9me2 levels were dramatically decreased upon G9a depletion (Figure 4C, lane 2). However, these levels in mESCs were not affected by mutations in the G9a ankyrin cage domain (Figure 4C, lanes 7 to 11). Surprisingly, a dramatic decrease in H3K9me2 levels was observed in mESCs harboring the G9aD905R mutation in the non-cage histone H3 binding surface of the ANK domain (Figure 4C, lanes 5 and 6). The H3K9me2 level was similar in cells expressing G9aD905R or lacking G9a altogether, despite the fact that the G9a D905R mutant is not catalytically dead when assayed *in vitro*[11]. It is well known that G9a exerts its histone methyltransferase activity in a heterodimeric complex with the G9a-like protein GLP [14]. In order to rule out the possibility that the impaired histone methylation observed in mESCs expressing G9aD905R (Figure 4C, lanes 5 and 6) was due to the fact that the D905R mutation in the G9a ANK domain abrogates the interaction between G9a and GLP, co-immunoprecipitation of transiently transfected FLAG-tagged full-length (WT or D905R mutant) mouse G9a and HA-tagged full-length mouse GLP was performed in COS-7 cell extracts. Co-immunoprecipitated GLP was detected when antibodies against FLAG were used for immunoprecipitation of wild-type G9a (Figure 4D, left panel, lane 4). These results indicate that G9a binding to GLP is not abrogated by the D905R mutation in the G9a ANK domain. Altogether, these findings reveal a novel role of the G9a ANK domain in global histone H3K9 methylation and support the hypothesis where the Asp905 residue of the G9a ANK domain non-cage histone H3 binding surface can potentially contribute to H3 substrate recognition by the SET domain.

### G9a, but not its ankyrin repeat histone binding surface, is required for the silencing of the embryonic stem cell-specific SSEA-1 surface marker during differentiation

Since G9a depletion or mutation of the G9a D905 ANK non-cage residue results in deficient *de novo* DNA methylation of *Oct3/4* during mESC differentiation, we further investigated whether this was accompanied by impaired transcriptional repression of *Oct3/4* in response to RA treatment. To answer this question, we performed an immunoblot analysis with an antibody against Oct3/4 protein using whole cell extracts derived from mESCs that were treated with RA for the indicated number of days (Figure 2A). Interestingly, the combined defects in both global H3K9 methylation levels and *de novo* DNA methylation observed in the G9a-null and G9a D905R mutant mESCs do not result in impaired repression of Oct3/4 protein expression in response to RA treatment (Figure 2A). Although the G9a protein levels appear to decrease after 8 days of RA treatment for the D905R mutant but not for the TT2 control (Figure 2A), this difference was not observed in repeat experiments. We further monitored by quantitative real-time PCR the expression of the *Oct3/4*, *Nanog*, *Klf4*, *Dppa3* and *Esrrb* early embryonic genes during mESC differentiation (Figure 2B). All WT, *G9a* knock-out and ANK mutant stable ESC lines displayed comparable mRNA levels of the above genes prior to RA treatment and the mRNA levels of these genes was similarly decreased in response to RA in all cell lines (Figure 2B). Altogether, these data show that G9a is not required for the transcriptional repression of several early embryonic genes in response to RA. The discrepancy between G9a protein levels (Figure 1B) which appear comparable amongst the parental TT2 and the constructed cell lines and G9a mRNA levels (Figure 2B), which are 50% lower in constructed cell lines compared to the parental cell line TT2 is due to the fact that the primers used to quantify G9a mRNA detect both G9a long and short isoforms which are both expressed at a 1:1 ratio in the TT2 cell line while cell lines constructed for the purpose of this study express solely the G9a long isoform (Figure 1B upper panel: 2 bands in TT2 lane in contrast to 1 band for all other constructed cell lines).

**Figure 5:**
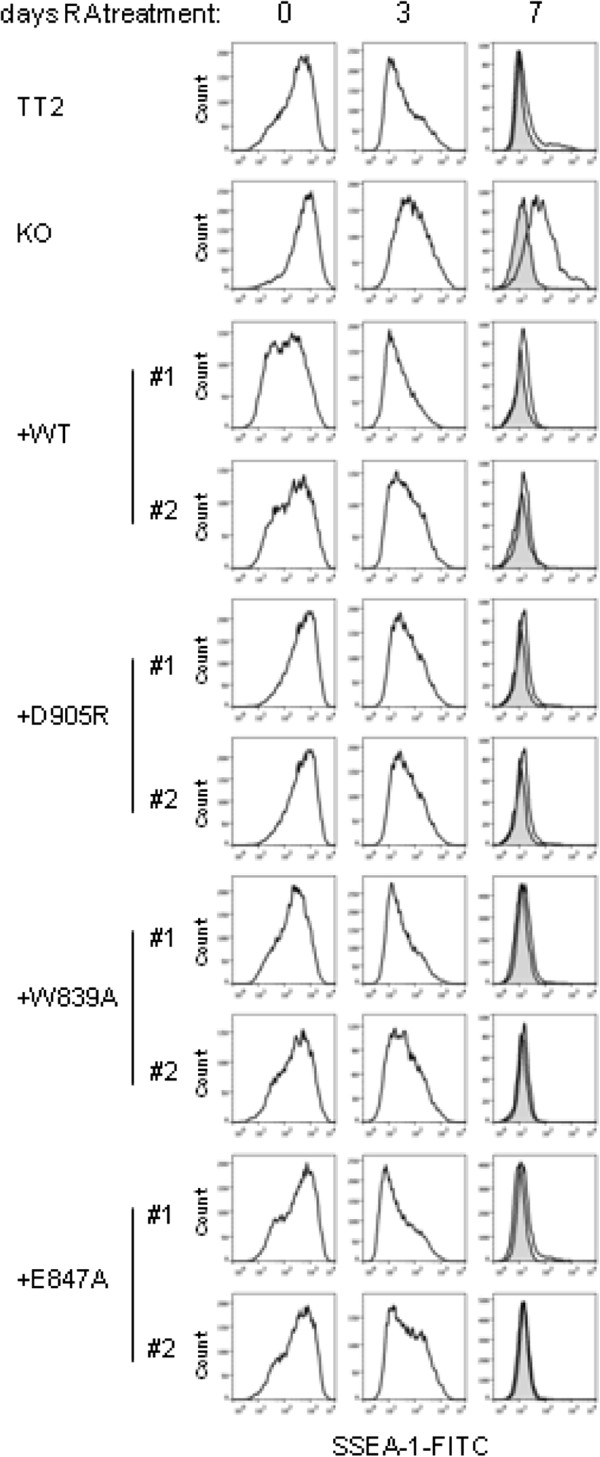
**G9a, but not the histone H3 binding surface of the ankyrin repeat (ANK) domain, is required for efficient repression of SSEA-1 expression during embryonic stem cell (ESC) differentiation induced by retinoic acid (RA).** Fluorescence-activated cell sorting (FACS) analysis of cell surface protein SSEA-1 expression in the indicated mESC lines that were untreated (0) or treated with RA for 3 or 7 days. Shaded histograms show nonspecific staining (obtained when staining exclusively with secondary antibody) and open histograms show specific SSEA-1 staining (staining with both primary and secondary antibodies).

The levels of the ESC-specific cell-surface marker SSEA-1 have been shown to inversely correlate with the degree of ESC differentiation. By flow cytometry, we observed that WT TT2 mESCs show high expression of SSEA-1 prior to RA treatment, and that these levels gradually decrease to background levels 7 days after the onset of RA treatment, as expected due to ESC differentiation (Figure 5). In contrast, *G9a*-null mESCs still retain partial SSEA-1 expression even 7 days after the onset of RA treatment, consistent with differentiation defects that have been attributed to the lack of G9a [15]. These results are in agreement with previous findings that G9a is essential for development and differentiation [16]. Interestingly, re-expression of WT as well as ankyrin cage and non-cage mutants of G9a rescued the impaired repression of SSEA-1 expression observed in the *G9a*-null ESCs (Figure 5). Indeed, all ESC lines analyzed, except the *G9a*-null line, show high expression of SSEA-1 prior to RA treatment and these levels gradually decrease to background levels 7 days after the onset of RA treatment. Altogether, these data suggest that domains or surfaces of G9a other than the histone H3 interaction surface of its ANK domain are required for shutting down expression of SSEA-1. In addition, the results indicate that G9a is only required for repression of a subset of the ESC genes that are turned off during the differentiation process.

## Discussion

### Distinct roles for the cage and non-cage regions of the G9a ankyrin repeat surface that binds K9-methylated histone H3

Our previous study demonstrated that the G9a ANK domain binds preferentially to histone H3 when it is modified by mono- or dimethylation at K9. In addition, we demonstrated that all of the ANK point mutations tested here disrupt binding to K9-monomethylated or -dimethylated histone H3. These mutations therefore defined a binding surface for histone H3 mono- or dimethylated at K9. The X-ray crystal structure indicated that the binding surface consists of a partial hydrophobic cage that encloses the dimethylamino moiety of the K9 residue of histone H3, and a surface outside of that cage that interacts with histone H3 residues 10 to 13 (Figure 1A, bottom panel). Surprisingly, the current study found that *de novo* DNA methylation of the *Oct3/4* promoter during RA-induced mESC differentiation was prevented only by the mutation of the D905 in the non-cage portion of histone H3 binding surface, while mutation of the cage residues had no effect on the *de novo* DNA methylation of *Oct3/4* (Figures 3B and 3D). These interesting results indicate that, while both the cage and non-cage residues are involved in binding of K9-methylated histone H3, the non-cage portion of the surface has a distinct function, which presumably involves binding of a yet unknown protein that mediates the role of G9a in *de novo* DNA methylation of the *Oct3/4* promoter. Although the percentage of DNA methylation at day 8 RA seen in the KO (Figure 3B; 26%) is higher than what was previously published by Epsztejn and colleagues, the percentage of DNA methylation we observed in the KO is consistently lower than both TT2 and +WT. We cannot rule out, however, that the phenotypes observed in the G9a D905R mutant may be due to a dominant gain of function rather than loss of function, and further studies would be required to investigate this. Importantly, our protein-protein interaction studies (Figures 4A and 4D) show that D905R mutant G9a maintains its ability to interact with both GLP and DNMT3A, therefore eliminating the possibility that the impaired DNA methylation could be due to absence of G9a-GLP complex formation or abrogated interaction with DNMT3A. Although the Asp905 residue of the G9a ankyrin domain is conserved in GLP, the phenotypes we have observed indicate that the Asp905 surface of the G9a ankyrin domain is non-redundant for its role in DNA methylation and cannot be compensated by the presence of wild-type GLP. This phenotype is in line with previous studies regarding G9a/GLP global H3K9 methylation: although both G9a and GLP conserved SET domains have histone methyltransferase activity, only the G9a SET domain (and not the GLP SET domain) catalytic function is required for H3K9 global methylation [10].

### Roles of the histone H3 binding surface and the DNMT3A binding surface of G9a in *de novo* DNA methylation of the *Oct3/4* promoter

The ability of G9a ANK domain to bind DNMT3A provides a potential mechanism for the G9a-dependent *de novo* DNA methylation of pluripotency genes such as *Oct3/4* during mESC differentiation; that is, G9a could recruit DNMT3A to the *Oct3/4* promoter. One way to test this hypothesis is to identify the DNMT3A binding surface of the G9a ANK domain and make point mutations that eliminate DNMT3A binding. However, we found that the G9a protein interaction surface that binds histone H3 methylated at K9 is not involved in the binding of DNMT3A by G9a. Thus, DNMT3A binds to a yet undefined surface of the G9a ANK domain. Furthermore, since the D905R mutation in the histone H3 binding surface eliminated G9a-dependent *de novo* DNA methylation of the *Oct 4* promoter without affecting the G9a-DNMT3A interaction, our results indicate that the ability of G9a to bind DNMT3A is not, by itself, sufficient to explain G9a-dependent *de novo* DNA methylation. Rather, while it still seems reasonable to assume that the binding of DNMT3A is relevant for G9a-dependent *de novo* DNA methylation, the ability of G9a to bind histone H3 or a yet unknown protein through the binding surface that includes D905 is also required.

### A novel role for the ankyrin repeat domain in G9a-dependent H3K9 methylation

Our studies have shown that G9a binding to H3K9me2 through the partial hydrophobic cage surface of the ANK domain is not required for the maintenance of global H3K9me2 levels. These results are surprising in the light of the fact that it has been thought that G9a binding to methyl-H3K9 might assist in propagating/spreading this methyl mark across genomic regions and thus might contribute to global cellular H3K9me2 levels. Data presented here do not support this hypothesis and leave open the question of the physiological relevance of G9a ANK binding to methyl-H3K9. In contrast, the D905R mutation not only prevented the G9a-dependent *de novo* DNA methylation during RA-induced differentiation of mESC, but also caused a dramatic reduction in global cellular levels of H3K9me2 in the undifferentiated mESC. Both of these phenotypes mirror that of mESC lacking G9a entirely, indicating that this ANK surface is critically important for both of these G9a functions. Importantly, the impaired DNA methylation observed in the G9a D905R ESCs is likely not due to defects in the formation of the G9a-GLP heteromeric complex since the D905R G9a mutation does not abrogate the interaction between G9a and GLP. Notably, although G9a-like protein (GLP) also has a SET domain harboring H3K9 methyltransferase activity, it is not surprising that the deficient H3K9me2 levels resulting from the G9a D905R mutation cannot be compensated by GLP since it has previously been shown that the catalytic activity of GLP is dispensable for H3K9 methylation *in vivo*[10].

As with *de novo* DNA methylation of the *Oct3/4* promoter, only the non-cage mutation D905R, but not any of the cage mutations, reduced cellular H3K9me2 levels. Thus, surprisingly these results indicate a critical role for the ANK domain in establishing and/or maintaining the global H3K9me2 levels. While the protein interaction by the non-cage ANK surface that is required to support H3K9 methylation by G9a has not been determined, the fact that this non-cage surface interacts with residues 10 to 13 of histone H3 in the previously published X-ray crystal structure suggests that this domain may somehow cooperate with the SET domain in recognizing and/or binding the H3 substrate for methylation by the SET domain. Although we did not detect ANK binding to unmethylated histone H3 *in vitro*[11], this does not rule out the possibility that the non-cage ANK surface could participate in an interaction with unmethylated histone H3 in conjunction with some other protein or G9a domain that binds unmethylated histone H3, such as the G9a SET domain. Moreover, while the SET domain alone is capable of methylating histone H3 *in vitro*, additional experiments are required to test whether the ANK domain and the non-cage histone H3 binding surface somehow enhance the enzymatic function of G9a.

### Roles of G9a in retinoic acid-induced differentiation of mouse embryonic stem cells

Previous studies demonstrated that G9a is required for mESC differentiation [15]. However, while *de novo* DNA methylation of the *Oct3/4* promoter fails to occur in *G9a*-null mESC, *Oct3/4* and *Nanog* gene expression is still repressed in these cells after RA treatment, and other specific molecular drivers and markers of the differentiation process that require G9a were not identified. By examining an expanded list of mESC-specific genes, we found that, in addition to *Oct3/4* and *Nanog*, G9a is also not required for repression of *Klf4*, *Esrrb* and *Dppa3* during RA-induced differentiation. Therefore, other histone modifiers (such as the PRC complex, for instance) and chromatin-remodeling proteins are most likely involved in the repression of these genes during ESC differentiation. In contrast, we found that repression of SSEA-1 is compromised by absence of G9a. Thus, G9a is required selectively for repression of mESC-specific genes, and SSEA-1 represents the first identified mESC-specific protein marker that requires G9a for its repression during RA-induced differentiation. This represents a valuable marker of G9a-mediated ESC differentiation defects that is both quantifiable and not lineage-specific. In addition, we found that none of the point mutations in the ANK surface that bind histone H3 prevented G9a-dependent repression of SSEA-1 expression, indicating that G9a domains outside of this histone H3-binding region of the ANK domain are responsible for repression of SSEA-1 by G9a during RA-induced differentiation. Furthermore, the fact that cell lines expressing D905R mutant G9a have compromised histone H3K9 and DNA methylation and yet retain the ability to repress SSEA-1 expression in response to RA suggests that histone H3K9 and DNA methylation are not required for early steps of ESC differentiation. However, these activities may be required to achieve full terminal differentiation.

## Conclusions

Our data validate previous findings suggesting that the G9a ANK domain is required for its role in *de novo* DNA methylation of *Oct3/4,* and we define a specific residue (Asp905) of the ANK domain as a key interaction surface likely involved in protein-protein interactions critical for G9a role in DNA methylation. Importantly, these findings open exciting avenues for investigating the protein(s) that interact(s) with G9a Asp905 in order to gain a better understanding of the precise molecular mechanisms of G9a in *de novo* DNA methylation. In addition, our data show that the G9a ANK domain methylhistone binding function is not required for its role in DNA methylation of Oct3/4 during ESC differentiation. Altogether, the results presented in this report advance our current understanding of the G9a ANK domain structure and distinct functional surfaces, revealing an unexpected role of the ANK domain both in global histone H3K9 methylation in undifferentiated ESCs and in *de novo* DNA methylation in a manner independent of the interaction between G9a and DNMT3A. Furthermore, these data collectively contribute to us gaining better insights into the molecular mechanisms (domains, surfaces and protein interactions) of G9a involved in ESC self-renewal and differentiation. Since epigenetic modifiers are increasingly being valued as promising targets for cancer drugs, it is important to investigate the role of G9a in ESC differentiation as a clearer understanding of this process would enable development of safer and more efficient reprogramming strategies for future therapeutic applications of stem cells, and shed light on fundamental questions concerning the establishment of cellular identity.

## Methods

### Mouse embryonic stem cell culture

TT2 line and *G9a*-null ES cells were provided by Dr. Yoichi Shinkai (Kyoto University, Kyoto, Japan). Undifferentiated ES cells were maintained in Dulbecco’s Modified Eagle’s medium (DMEM, GIBCO) supplemented with 13% (vol/vol) Stem Cell Qualified FBS (Gemini), 100 μM nonessential amino acids (GIBCO), 100 μM 2-mercaptoethanol (Sigma) and 10^3^ U/ml leukemia inhibitory factor (LIF; Millipore) at 37°C, and 5% CO2. ESCs were seeded on gelatin (Millipore)-coated dishes. For retinoic-acid (RA)-induced differentiation, cells were cultured in the presence of 1 μM *all-trans*-RA (Sigma) without LIF.

### Generation of G9a-mutant embryonic stem cells

To generate stable cell lines expressing wild-type (WT) or mutant G9a, the pHRCMVpuro-Sin8 vector containing a puromycin resistance cassette was introduced simultaneously with a threefold excess molar ratio of the G9a expression vector into the *G9a*-null ES cells by either standard electroporation or by nucleofection. Nucleofection (1 μg of plasmid DNA per 10^6^ cells) was performed using the Amaxa 4-D Nucleofector System (Lonza) with the P3 Primary Cell 4-D Nucleofector X Solution and the CG-104 program. Plasmid DNA used for nucleofection was purified using the Endotoxin-free Plasmid Maxiprep Kit (Qiagen) according to the manufacturer’s instructions. The stable transfectant clones expressing mutant or WT G9a were selected in ESC medium containing puromycin (1 μg/ml). All mutations were confirmed in the selected cell clones by sequencing.

### Plasmids

The following mammalian expression vectors were described in previous publications: pSG5-FLAG encoding full-length mouse G9a protein, including wild-type, ankyrin mutants (D905R, E870R, W839A, E847A and W877A) and SET mutant (H1166K) [11]; pcDNA3.1-mDNMT3A [17]; and pHRCMVpuro-Sin8 [18]; pSG5-HA encoding full length mouse G9a and a deletion mutant (ΔANK) lacking the ankyrin repeat domain (amino acids 734 to 934) [19]. The GLP expression vector pSG5-FLAG GLP (full-length, wild-type) was generated by PCR.

### Antibodies used for immunoblot

The following antibodies were purchased and used for this study: antibody to G9a (PP-A8620A-00) from R&D Biosystems; antibody to DNMT1 (IMG-261A) from Imgenex; antibody to HA (11867431001) from Roche; antibodies to H3K9me2 (ab1220), DNMT3A (ab2850), and DNMT3B (ab2851) from Abcam; antibodies to Oct3/4 (sc-8629), and H3 (sc-10809) from Santa Cruz Biotechnology; antibodies to FLAG (F1804), and GAPDH (G9545) from Sigma; antibodies to Sox2 (AB5603), and Nanog (AB5731) from Millipore. Immunoblot detection was performed by chemiluminescence using Super Signal West Dura (Thermo Scientific) for proteins with low expression levels and HyGLO (Denville Scientific) for all other proteins according to the manufacturer’s instructions. Immunoblots were performed on whole cell extracts (RIPA buffer) or Acid Histone Extracts (http://www.abcam.com/protocols/histone-extraction-protocol-for-western-blot).

### Real-time RT-qPCR analysis

Total RNA was isolated using TRIzol (Invitrogen) according to the manufacturer’s instructions. Reverse transcription was performed by using iScript Advanced cDNA Synthesis Kit for RT-qPCR (BioRad) according to the manufacturer’s instructions by using 5 μg of total RNA as template. qPCR amplification was performed on a Roche LightCycler 480 by using SYBR Green I Master Mix (Roche) and the following PCR primers: G9a.Fwd (5′- AGCTTCGGAACAAAGAAGGAG -3′) and G9a.Rev (5′- ACAGGGGATGGGTACATTCTC -3′); UBC.Fwd (5′- CAGCCGTATATCTTCCCAGACT -3′) and UBC.Rev (5′- CTCAGAGGGATGCCAGTAATCTA -3′); Oct3/4.Fwd (5′- CTGAAGCAGAAGAGGATCACC -3′) and Oct3/4.Rev (5′- GCCGCAGCTTACACATGTTC -3′); Nanog.Fwd (5′- CAGCCTCCAGCAGATGCAAG -3′) and Nanog.Rev (5′- AATCAGACCATTGCTAGTCTTC -3′); Klf4.Fwd (5′- TCTCAAGGCACACCTGCGAAC -3′) and Klf4.Rev (5′- GGTAGTGCCTGGTCAGTTCATC -3′); Esrrb.Fwd (5′- CGCAAGAGCTACGAGGACTG -3′) and Esrrb.Rev (5′- GGTAGCCAGAGGCAATGTCC -3′); and Dppa3.Fwd (5′- AGTCTACGGAACCGCATTGC -3′) and Dppa3.Rev (5′- GCTATAGGGTCTTTCAGCACCG -3′).

### Bisulfite gDNA sequencing

Genomic DNA was isolated from ESC lines that were untreated or treated with RA for 8 days using the DNeasy Blood and Tissue Kit (Qiagen) according to the manufacturer’s instructions. Bisulfite treatment of the genomic DNA was performed using the EZ DNA Methylation Kit (Zymo Research) according to the manufacturer’s instructions. PCR was performed to amplify the *Oct3/4* promoter with the following primers: Oct3/4.bs.F (5′- TGGGTTGAAATATTGGGTTTATTT -3′) and Oct3/4.bs.R (5′- CTAAAACCAAATATCCAACCATA -3′). PCR products were subcloned into pCR 2.1-TOPO vector using the TOPO TA Cloning Kit (Invitrogen) and sequencing was performed using the M13R primer that localizes to the vector backbone.

### Transfection co-immunoprecipitation

COS- 7 cells were transiently transfected by using Lipofectamine 2000 (Invitrogen) with 5 μg of plasmid encoding G9a and 1 μg of plasmid encoding DNMT3A or, alternatively, 4 μg of plasmid encoding GLP. At 72h after transfection, cell extracts were prepared in RIPA buffer with Nonidet P-40 and sodium deoxycholate replaced by Triton X-100 (50 mM Tris- HCl (pH 8.0), 150 mM NaCl, 1% Triton X-100, and 1 mM EDTA). Antibodies used were the same as those indicated for immunoblots.

### Fluorescence-activated cell sorting analysis and cell sorting

Cells were trypsinized, washed once in PBS, and resuspended in PBS; dead cells were stained using the LIVE/DEAD Fixable Dead Cell Stain Kit (Invitrogen) according to the manufacturer’s instructions. Next, cells were fixed in 2% paraformaldehyde (PFA) for 15 min at room temperature, washed with PBS and stained with anti-SSEA-1 mouse monoclonal IgM antibody (mc-480, University of Iowa, Developmental Studies Hybridoma Bank, 1:400) at 37°C for 20 min. After a PBS wash, the cells were labeled with fluorescein isothiocyanate (FITC)-conjugated goat anti-mouse IgM (Millipore, 1:200) for 15 min at 37°C. After one PBS wash, stained cells were resuspended in PBS containing 1% FBS and 3% BSA, and at least 10,000 events of healthy cells were analyzed with a CyAN Analyzer (Beckman Coulter) and FlowJo ver.10 software (Tree Star, Ashland, USA). Control cells were not treated with primary antibody.

## Abbreviations

ANK: ankyrin repeat; ESC: embryonic stem cell; KO: knock-out; HA: human influenza hemagglutinin; WT: wild-type; mRNA: messenger RNA; FACS: fluorescence-activated cell sorting; H3K9: histone H3 lysine K9; RA: retinoic acid; SSEA-1: stage-specific embryonic antigen 1.

## Competing interests

The authors declare that they have no competing interests.

## Authors’ contributions

DB supervised the project, conceived strategies, designed and performed experiments, analyzed data and wrote the paper; BHL. performed experiments and analyzed data; LG performed experiments and analyzed data; DSG. performed experiments and analyzed data; M.R.S. supervised the project and wrote the paper. All authors read and approved the final manuscript.
